# Exploring the Shared Diagnostic Biomarkers and Molecular Mechanisms Related to Mitochondrial Dysfunction in Inflammatory Bowel Disease and Rheumatoid Arthritis

**DOI:** 10.3390/cimb48010089

**Published:** 2026-01-16

**Authors:** Lijiao Cui, Shicai Ye, Zhiwei Gu, Guixia Zhang, Tingen Chen, Yu Zhou, Caiyuan Yu

**Affiliations:** 1 Laboratory of Gastroenterology, Affiliated Hospital of Guangdong Medical University, Zhanjiang 524000, China; gdmuclj2020@126.com (L.C.); yeshicai@gdmu.edu.cn (S.Y.); 2 Department of Gastroenterology, Affiliated Hospital of Guangdong Medical University, Zhanjiang 524000, China; 13413690961@163.com (Z.G.); zgxzh2021@126.com (G.Z.); cte391215@163.com (T.C.)

**Keywords:** inflammatory bowel disease, rheumatoid arthritis, mitochondrial dysfunction, DUSP6, PDIA4, immune infiltration

## Abstract

Inflammatory bowel disease (IBD) and rheumatoid arthritis (RA) are chronic inflammatory diseases that share immune dysregulation and mitochondrial dysfunction. Understanding the molecular mechanisms linking these diseases to mitochondrial dysfunction is crucial for developing novel diagnostic and therapeutic strategies. Datasets related to IBD and RA were obtained from the Gene Expression Omnibus database. Differentially expressed mitochondrial dysfunction-related genes (MDRGs) were identified using differential expression analysis. Weighted gene co-expression network analysis was performed to identify crosstalk genes (CGs). Logistic regression and support vector machine (SVM) models were constructed using least absolute shrinkage and selection operator regression to identify hub genes. Additionally, the differential expression and diagnostic value of the hub genes were verified using quantitative reverse transcriptase–polymerase chain reaction and validation sets. Finally, immune infiltration analysis was conducted to assess the role of immune cells in IBD and RA. A total of 87 CGs associated with mitochondrial dysfunction were identified between IBD and RA, among which *PDIA4* and *DUSP6* were identified as hub genes. Twenty proteins, including ERO1A, MAPK7, and P4HB, were identified as key proteins that interacted with PDIA4 and DUSP6. The area under the curve (AUC) of the ROC curves for IBD and RA based on the *DUSP6* and *PDIA4* diagnostic models were 0.664 and 0.856, respectively. The qRT-PCR results indicated that *PDIA4* and *DUSP6* were overexpressed in IBD and RA. Seven immune cell types, including activated B cells, activated dendritic cells, and eosinophils showed significant differences in the IBD and RA groups. Our findings highlight the close association between IBD, RA, and mitochondrial dysfunction. *PDIA4* and *DUSP6* may serve as potential biomarkers of mitochondrial dysfunction in patients with IBD and RA.

## 1. Introduction

Inflammatory bowel disease (IBD) represents a spectrum of chronic, relapsing inflammatory conditions affecting the gastrointestinal tract, predominantly comprising Crohn’s disease and ulcerative colitis. Although its exact pathogenesis remains unclear, current evidence suggests that IBD arises from a complex interplay of multiple factors, including genetic predisposition, environmental triggers, gut microbiota dysbiosis, and dysregulated immune inflammatory responses [1]. Clinically, IBD manifests not only with intestinal symptoms but also with extraintestinal manifestations (EIMs), which occur in approximately one-third of patients with IBD, predominantly affecting the joints, skin, eyes, and hepatobiliary system [2]. Among these EIMs, articular involvement is the most common, with epidemiological studies indicating that 20–30% of patients with IBD develop arthropathy [3]. Rheumatoid arthritis (RA) is a chronic inflammatory autoimmune disease primarily affecting the joints. Its pathogenesis is similar to that of IBD, primarily involving genetic susceptibility, environmental influences, and immune dysregulation [4].

The relationship between IBD and RA is particularly intriguing, as both conditions share similar pathogenic mechanisms, including immune dysregulation and genetic susceptibility. Patients with IBD have an increased risk of developing extraintestinal manifestations, including arthritis, which may resemble RA [5]. Conversely, patients with RA may experience gastrointestinal symptoms that mimic IBD, complicating diagnosis and management [6]. Recent studies suggest a close association, and possibly a causal relationship, between IBD and RA [7,8]. Understanding their interplay and overlapping phenotypes is crucial to develop targeted therapeutic therapies and improve patient outcomes.

Mitochondrial dysfunction is a pathological condition characterized by abnormalities in energy production, metabolism, and physiological functions [9]. It is implicated in various diseases, including IBD and RA, affecting millions of people worldwide [10]. Studies have identified multiple hallmarks of mitochondrial dysfunction, including impaired ATP production and elevated oxidative stress, in the intestinal mucosa of patients with IBD [11]. Further research highlights its pivotal role in IBD pathogenesis and recurrence, contributing to epithelial barrier disruption, immune dysregulation, and amplified inflammatory signaling [12]. Similarly, mitochondrial dysfunction has also been implicated in the pathogenesis of RA [13]. Structurally and functionally intact mitochondria are essential for maintaining homeostasis in synovial fibroblasts and chondrocytes [14]. Disruption of mitochondrial integrity impairs the survival and function of immune cells involved in RA pathogenesis, leading to immune dysregulation and promoting the development and progression of RA [15].

Although accumulating evidence supports a significant association between IBD and RA, and research increasingly implicates mitochondrial dysfunction in their pathogenesis, the shared, network-level molecular mechanisms driven by mitochondrial dysfunction remain largely unexplored. Specifically, it is unclear whether there exists a common set of core genes and pathways that mediate the crosstalk between these two diseases through mitochondrial dysregulation.

To bridge this knowledge gap, we conducted a systematic analysis using datasets from public databases on these two diseases and mitochondrial dysfunction. Weighted gene co-expression network analysis (WGCNA) was used to identify the co-expression modules and shared crosstalk genes (CGs) between IBD and RA. This network-based approach is pivotal as it transcends the study of isolated molecules, enabling the systemic discovery of interconnected gene modules that may collectively drive disease comorbidity. A key advantage of this approach is its capacity to bridge different biological layers—connecting upstream cellular processes like mitochondrial dysfunction with downstream phenotypic outcomes such as inflammatory signaling and immune dysregulation—thereby providing a holistic view of the shared pathophysiology between IBD and RA. In this study, ‘CGs’ refer to the high-confidence gene set obtained by intersecting the disease-associated, mitochondrial dysfunction-related genes (MDRGs) from both co-expression network and differential expression analyses of IBD and RA, representing key molecular candidates for shared pathophysiology. Subsequently, we performed functional enrichment, constructed diagnostic models to identify hub genes (*PDIA4* and *DUSP6*), and validated their association with immune cell infiltration. This integrative, network-based approach aims to elucidate the shared mitochondrial dysfunction-related architecture underlying IBD-RA comorbidity.

## 2. Materials and Methods

### 2.1. Data Acquisition and Preprocessing

IBD (GSE75214 and GSE179285) and RA (GSE89408 and GSE17755) datasets were downloaded from the GEO database (https://www.ncbi.nlm.nih.gov/geo/ (accessed on 25 November 2024)). Samples of GSE75214 and GSE179285 were sourced from the colon, while the samples of GSE89408 were from synovial tissues, and those of GSE17755 were from peripheral blood. GSE75214 and GSE89408 served as experimental sets, whereas GSE179285 and GSE17755 were validation sets. Detailed dataset information is presented in Table 1. The four datasets were standardized using the R package limma (Version 3.58.1) [16], with probe annotation and normalization performed (Supplementary Figure S1). Mitochondrial dysfunction-related genes (MDRGs) were collected from the GeneCards database [17] (https://www.genecards.org/ (accessed on 26 November 2024)) and relevant literature indexed in PubMed (https://pubmed.ncbi.nlm.nih.gov/ (accessed on 26 November 2024)) [18].

### 2.2. Differential Genes Expression Analysis

To identify the differentially expressed genes (DEGs) in IBD and RA, differential expression analyses were performed on the GSE75214 and GSE89408 datasets using the R package limma (version 3.58.1) [16]. DEGs were defined based on a threshold of |logFC| > 1 and an adjusted *p*-value < 0.05, with *p*-value correction performed using the Benjamini–Hochberg (BH) method. The results of the difference analyses were visualized as volcano plots using the R package ggplot2 (version 3.4.4).

To obtain mitochondrial dysfunction-related DEGs (MDRDEGs) in IBD and RA, all identified DEGs in IBD or RA were intersected with MDRGs and Venn diagrams were generated to illustrate the overlap. The intersecting genes were classified as IBD-related MDRDEGs (IBD-MDRDEGs) or RA-related MDRDEGs (RA-MDRDEGs). Additionally, heatmaps were created using the R package pheatmap (version 1.0.12) to highlight the top 20 IBD- and RA-MDRDEGs.

### 2.3. WGCNA and Identification of CGs

To identify co-expression module that align with scale-free topology in IBD and RA, WGCNA [19] was performed on the top 90% of DEGs from the GSE89408 and GSE75214 using the R WGCNA package (version 1.73) [20]. The variances of all genes in these datasets were calculated, and the top 90% most variable genes were selected. The following parameters were set for module detection: a minimum module size of 200 genes, a scale-free topology fitting index of 0.85, a module merging threshold of 0.2, and minimum distance of 0.2. Then, the modules (|*r*| > 0.3 were screened, and the genes in these modules were intersected with MDRGs. Venn diagrams were separately plotted. All intersection genes obtained from the different modules were IBD-WGCNA-MDRGs and RA-WGCNA-MDRGs. Finally, the intersections of IBD-WGCNA-MDRGs, RA-WGCNA-MDRGs, IBD-MDRDEGs, and RA-MDRDEGs were obtained, and the resulting genes constituted the CGs.

### 2.4. Enrichment Analysis of CGs

Gene Ontology (GO) [21] and Kyoto Encyclopedia of Genes and Genomes (KEGG) [22] analyses of CGs were performed using the R package of clusterProfiler (version 4.10.0) [23]. To evaluate whether different pathways were enriched across different samples, the c2.cp.v2023.2.Hs.symbols.gmt gene set was obtained from the Molecular Signatures Database (MSigDB) [24]. Gene set variation analysis (GSVA) [25] was then performed on all genes in GSE7521 and GSE89408 using the R package GSVA (version 1.50.0). The screening criteria of GO, KEGG, and GSVA were adjusted to *p* < 0.05, and the Benjamini–Hochberg (BH) *p*-value correction method was used.

### 2.5. Screening of Potential Diagnostic Biomarkers in CGs

To determine the diagnostic value of CGs in IBD and RA, logistic regression analysis was performed on the CGs with *p* < 0.05 as the selection criterion. An SVM was constructed using the SVM algorithm [26], whereas least absolute shrinkage and selection operator (LASSO) regression analysis was conducted using the R package glmnet (version 4.1-8) [27], with parameters set to seed = 500 and family = “binomial”. The LASSO regression results represented diagnostic models for IBD and RA, identifying CGs as IBD-key genes and RA-key genes. These results were visualized using diagnostic model diagrams and variable trajectory plots. Next, the IBD-key genes, RA-key genes, were intersected with all genes in the GSE179285 and GSE17755 datasets to identify hub genes. Finally, the LASSO risk score was calculated based on the risk coefficients from the LASSO regression analysis using the following formula:

$$riskScore=\sum_{i}Coefficient\left({gene}_{i}\right)\times mRNA\;Expression({gene}_{i})$$

### 2.6. Validation of Diagnostic Models Based on Identified Hub Genes

The R package rms (Version 6.7-1) was used to draw a nomogram [28] based on the results of the logistic regression analysis to illustrate the relationship among hub genes. The R package ggDCA (version 1.1) [29] was used to conduct decision curve analysis (DCA), a method for evaluating clinical prediction models, diagnostic tests, and molecular markers, to assess hub genes in IBD and RA datasets. Subsequently, the R package pROC [30] (version 1.18.5) was used to depict the ROC curves and calculate the area under the curve (AUC) to evaluate the diagnostic efficacy of the LASSO risk score in predicting the occurrence of IBD and RA.

### 2.7. Construction of PPI Network and Regulatory Network

The PPI network was constructed to predict functionally similar hub genes using the GeneMANIA database [31] (https://genemania.org/ (accessed on 6 December 2024)), which integrates data from various public databases, such as GO, KEGG, Reactome, BioGRID, and STRING. Transcription factors (TFs) regulate gene expression by interacting with hub genes during the post-transcriptional stage. By searching for TFs in the ChIPBase database [32] (http://rna.sysu.edu.cn/chipbase/ (accessed on 6 December 2024)), we analyzed the regulatory effects of these TFs on hub genes and visualized the mRNA-TF regulatory network using the Cytoscape software version 3.9.1 (Cytoscape Consortium, San Diego, CA, USA) [33]. In addition, to explore the relationship between the hub genes and miRNAs, we utilized the StarBase v3.0 database [34] (https://starbase.sysu.edu.cn/ (accessed on 6 December 2024)) to identify miRNAs associated with hub genes. Subsequently, the mRNA-miRNA regulatory network was visualized using Cytoscape software.

### 2.8. ROC Curve Analysis of Hub Genes

To explore differences in the expression of hub genes in IBD and RA, we plotted group comparison graphs based on the expression levels of their respective hub genes. Finally, the R package pROC [30] (version 1.18.5) was employed to generate ROC curves for the hub genes and calculate the AUC to evaluate the diagnostic efficacy of hub gene expression levels on the occurrence of IBD or RA. The AUC of the ROC curve generally ranged from 0.5 to 1, with values closer to 1 indicating superior diagnostic performance.

### 2.9. Analysis of Immune Cell Infiltration

Single-sample gene set enrichment analysis (ssGSEA) [35] was used to label various infiltrating immune cell types, calculate the relative abundance of each type of immune cell infiltration in each sample, and form an immune cell infiltration matrix of the validation sets GSE179285 and GSE17755. The R package ggplot2 (version 3.4.4) was used to create grouped comparison diagrams to illustrate differences in the expression of immune cells in IBD and RA. Immune cells showing significant differences between the two groups were screened for subsequent analyses. The Spearman algorithm was used to analyze the correlation between hub genes and immune cells. The correlation analysis results were visualized using the R packages pheatmap (version 1.0.12) and ggplot2 (version 3.4.4).

### 2.10. Cell Culture and Construction of Inflammation Model

The NCM460, RA-FLS, and THP-1 human cell lines were sourced from the Cell Resource Center of the Chinese Academy of Sciences (Shanghai, China). NCM460 and THP-1 cells were cultured in RPMI-1640 medium (Gibco; Thermo Fisher Scientific, Waltham, MA, USA) supplemented with 10% fetal bovine serum (FBS) (Thermo Fisher Scientific, Waltham, MA, USA) and 1% penicillin-streptomycin, and RAFLS cells were cultured in DMEM/F-12 (1:1) (Thermo Fisher Scientific, Waltham, MA, USA) supplemented with 10% FBS and 1% penicillin-streptomycin-glutamine (MCE, Shanghai, China). All cells were cultured in a humidified incubator at 37 °C and 5% CO_2_. When the cells reached optimal growth conditions, they were seeded at a density of 2 × 10^5^ cells per well in a 12-well plate and cultured for 24 h. Subsequently, the cells in the 12-well plate were equally divided into two groups, namely inflammatory group (*n* = 6) and control group (*n* = 6). The inflammatory groups of the three cell lines were treated with lipopolysaccharide (LPS) at concentrations of 10 µg/mL to establish inflammatory cell models and control groups without LPS treatment. After 24 h of culture, the cells were harvested for downstream applications.

### 2.11. Real-Time Quantitative Reverse Transcriptase-Polymerase Chain Reaction (qRT-PCR)

Total RNA was extracted from cellular samples using TRIzol reagent (Invitrogen, Carlsbad, CA, USA), followed by reverse transcription into cDNA with the PrimeScript^®^ RT Master Mix Perfect Real Kit (Takara, Kusatsu, Japan). qRT-PCR was conducted using TB Green^®^ Premix Ex Taq™ II (Tli RNaseH Plus) kits (Takara, Kusatsu, Japan) on a LightCycler 480II (Roche, Basel, Switzerland). The relative expression level of dual-specificity phosphatase 6 (*DUSP6*) and protein disulfide isomerase A4 (*PDIA4*) mRNA was calculated using the 2^−ΔΔCt^ method, with *GAPDH* as the reference gene. The primers for amplification were synthesized by Tsingke Biotech (Guangzhou, China), with sequences as follows: *DUSP6* forward, 5′-CTTGGACGTGTTGGAGGAAT-3′; *DUSP6* reverse 5′-AATGGCCTCAGGGAAAAACT-3′; *PDIA4* forward, 5′-GAGAGTGGGGAGGATGTCAA-3′; *PDIA4* reverse, 5′-ACTGGCTGGGATTTGATGAC-3′; *GAPDH* forward, 5′-GGGTGTGAACCATGAGAAGT-3′; *GAPDH* reverse, and 5′-CAGTGATGGCATGGACTGTG-3’.

### 2.12. Statistical Analysis

All data processing and analyses were conducted using R software (Version 4.2.2). For comparisons of continuous variables between the two groups, the statistical significance of normally distributed variables was estimated using the independent Student’s *t*-Test, unless otherwise specified. The Mann–Whitney U Test (Wilcoxon rank-sum test) was used for non-normally distributed variables. The Kruskal–Wallis test was used for comparison involving three or more groups. Spearman’s correlation analysis was used to calculate the correlation coefficients between the different molecules. All statistical *p* values were two-sided if not specified, and a *p* value < 0.05 was considered statistically significant.

### 2.13. Technology Roadmap

**Figure 1:**
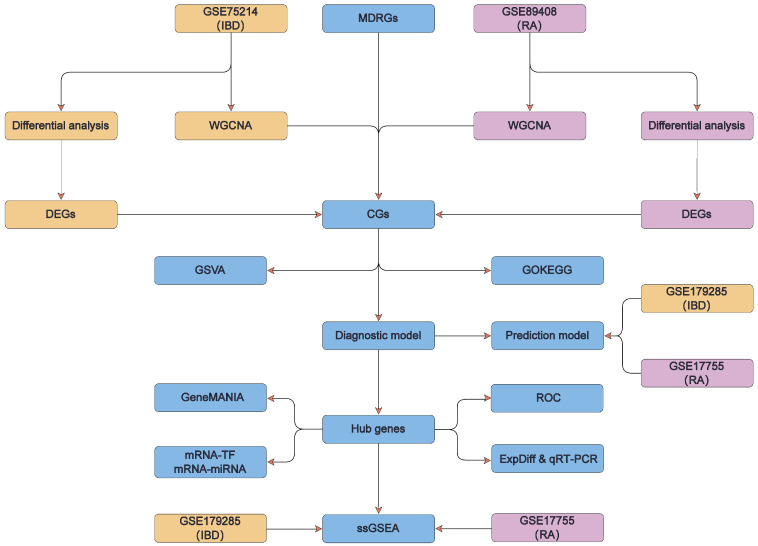
Technology roadmap of this study. IBD, inflammatory bowel disease; RA, rheumatoid arthritis; MDRGs, mitochondrial dysfunction related genes; DEGs, differentially expressed genes; WGCNA, weighted gene co-expression network analysis; CGs, crosstalk genes; GO, Gene Ontology; KEGG, Kyoto Encyclopedia of Genes and Genomes; GSVA, gene set variation analysis; TF, transcription factor; ssGSEA, single-sample gene-set enrichment analysis.

The overall design approach of this study is illustrated in Figure 1.

## 3. Results

### 3.1. DEGs in IBD/RA Related to Mitochondrial Dysfunction

**Figure 2:**
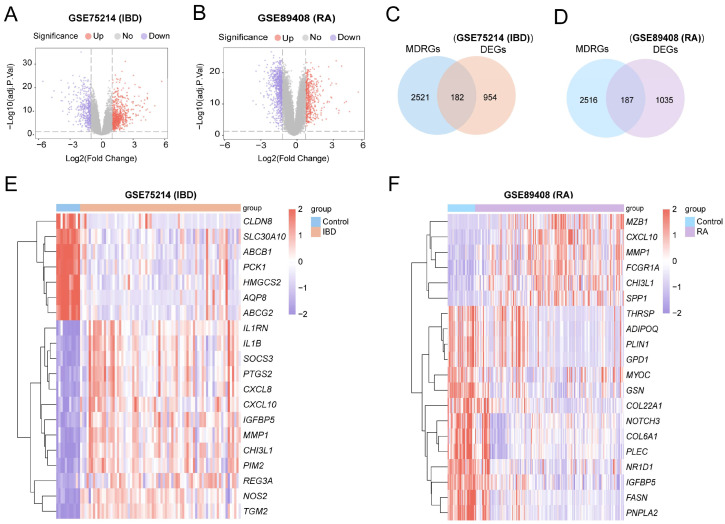
DEGs analysis in IBD and RA. (**A**,**B**) Volcano plots of DEGs in GSE75214 and GSE89408. (**C**,**D**) Venn diagrams of MDRGs and DEGs in GSE75214 and GSE89408. (**E**,**F**) Heatmaps of MDRDEGs in GSE75214 and GSE89408.

In GSE75214, 1136 IBD-DEGs were identified, with 724 upregulated and 412 downregulated genes, and a volcano plot was generated (Figure 2A). In GSE89408, 1222 RA-DEGs were identified, with 469 upregulated and 753 downregulated genes, and a volcano plot was generated (Figure 2B). A total of 2703 MDRGs were obtained by combining the GeneCards database, published literature data, and deduplication (Supplementary Table S1). A total of 182 IBD-MDRDEGs (Supplementary Table S2) and 187 RA-MDRDEGs (Supplementary Table S3) were obtained after the DEGs intersected with MDRGs, and Venn diagrams were drawn (Figure 2C,D). Additionally, heat maps were drawn to display the specific analysis results for the top 20 MDR-DEGs in GSE75214 and GSE89408 (Figure 2E,F).

### 3.2. WGCNA and the Acquisition of CGs of IBD and RA

**Figure 3:**
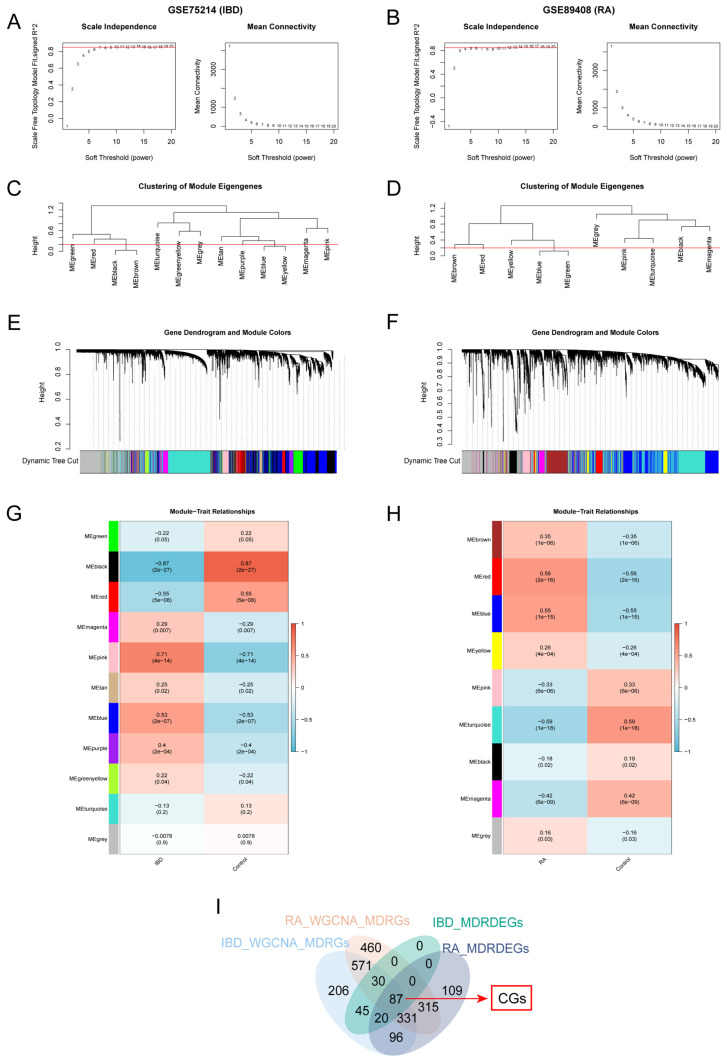
WGCNA of IBD and RA. (**A**,**B**) Determination of the best soft threshold in GSE75214 and GSE89408. (**C**,**D**) Clustering trees of module eigengenes in GSE75214 and GSE89408. (**E**,**F**) Genes clustering dendrograms in GSE75214 and GSE89408. (**G**,**H**) Relationships between module eigengenes and traits in disease group and control group, with correlation coefficients and *p* value presenting in each module. (**I**) Venn diagram for intersecting genes of IBD-WGCNA-MDRGs, RA-WGCNA-MDRGs, IBD-MDRDEGs and RA-MDRDEGs. |*r*| < 0.3, weak or no correlation, 0.3< |*r*| < 0.5, weak correlation, 0.5 < |*r*| < 0.8, moderate correlation, |*r*| > 0.8, strong correlation. Red represents positive correlation, blue represents negative correlation.

According to WGCNA, the optimal soft threshold was 10 in GSE75214 and 12 in GSE89408 (Figure 3A,B) when the fitting index was 0.85. Based on the optimal soft threshold, genes with the top 90% variance were clustered and labeled with grouping information using clustering trees (Figure 3C,D). Based on the similarity between modules, nine modules were clustered in GSE89408 and 11 modules were clustered in GSE75214 (Figure 3E,F). The correlations between module eigengenes and groups were calculated and visualized using heat maps (Figure 3G,H). When the standard of clustering modules was |*r*| > 0.3, five modules in GSE75214 and six modules in GSE89408 were screened (Supplementary Figure S2). Ultimately, 1794 RA-WGCNA-MDRGs (Supplementary Table S4) and 1386 IBD-WGCNA-MDRGs (Supplementary Table S5) were obtained. More importantly, 87 genes (Supplementary Table S6) were obtained by intersecting IBD-WGCNA-MDRGs, RA-WGCNA-MDRGs, IBD-MDRDEGs, and RA-MDRDEGs, which were defined as CGs (Figure 3I).

### 3.3. GO and KEGG Enrichment Analysis for CGs

**Figure 4:**
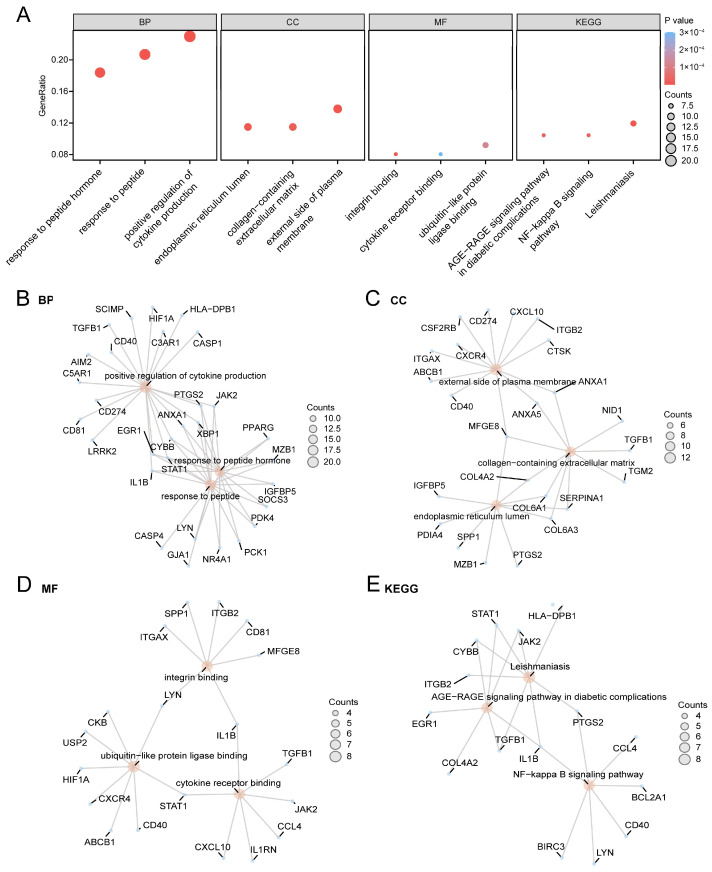
GO and KEGG enrichment analysis for CGs. (**A**) Bubble diagrams of GO and KEGG enrichment analysis results of CGs. (**B**–**E**) Network diagrams of GO and KEGG enrichment analysis results of CGs: BP (**B**), CC (**C**), MF (**D**) and KEGG (**E**).

GO and KEGG enrichment analyses were performed for the 87 CGs (Supplementary Table S7). The analysis revealed that CGs were mainly enriched in biological processes (BP), such as positive regulation of cytokine production, response to peptides, and response to peptide hormones; in cellular components (CC), such as the external side of the plasma membrane, endoplasmic reticulum lumen, and collagen-containing extracellular matrix; and in molecular functions (MF), such as integrin binding, ubiquitin-like protein ligase, and cytokine receptor binding. Furthermore, these CGs were also enriched in biological pathways including leishmaniasis, AGE-RAGE signaling pathway in diabetic complications, NF-κB signaling pathway and other biological KEGG. The results of GO and KEGG enrichment analyses were shown as bar graphs (Figure 4A). Network diagrams of BP, CC, MF, and KEGG were drawn according to the GO and KEGG enrichment analyses (Figure 4B–E).

### 3.4. GSVA for IBD and RA

**Figure 5:**
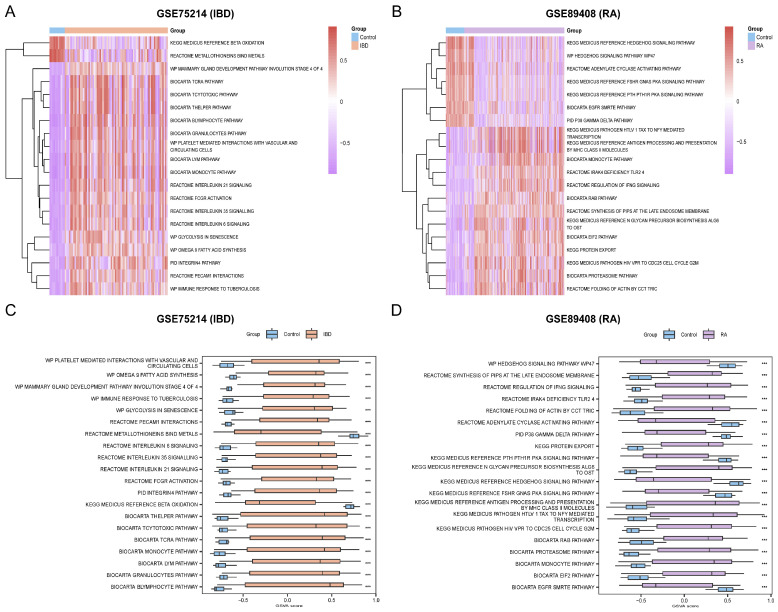
GSVA results in IBD and RA. (**A**) Heatmap of GSVA results between IBD group and control group in GSE75214. (**B**) Heatmap of GSVA results between RA group and control group in GSE89408. (**C**) Group comparison map of GSVA results between IBD group and control group in GSE75214. (**D**) Group comparison map of GSVA results between RA group and control group in GSE89408. *** *p* < 0.001.

GSVA for IBD (Supplementary Table S8) and RA (Supplementary Table S9) was performed on all genes in the GSE75214 and GSE89408 datasets. The top 20 pathways in IBD and RA were analyzed and visualized using heat maps (Figure 5A,B). The results of GSVA showed that the immune response to tuberculosis, PECAM1 interactions, and other identified top 20 pathways were statistically significant in IBD (*p* < 0.05) (Figure 5A), whereas the folding of actin by CCT tric, the biocarta proteasome pathway, and other identified top 20 pathways were significantly different in RA (*p* < 0.05) (Figure 5B). Subsequently, the differences were verified using the Mann–Whitney U test, and group comparison maps were drawn to display the results (Figure 5C,D).

### 3.5. Construction of Diagnostic Model for IBD and RA

**Figure 6:**
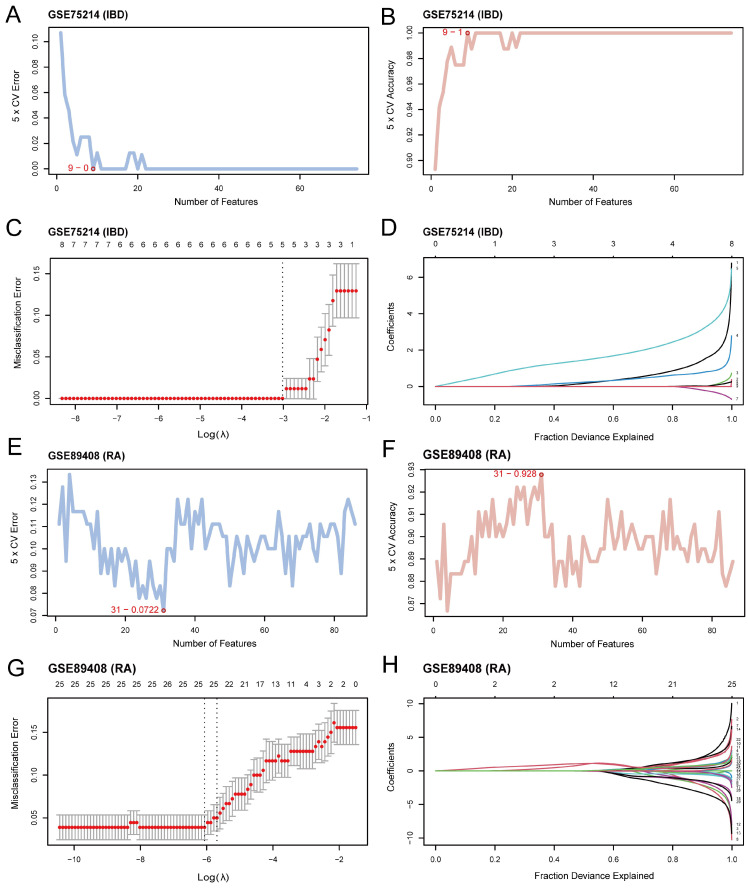
Diagnostic model for IBD and RA. (**A**,**B**) Visualization of the number of genes with the lowest error rate (**A**) and the number of genes with the highest accuracy (**B**) based on SVM algorithm in GSE75214 dataset. (**C**,**D**) LASSO regression analysis in GSE75214 dataset. Diagnostic model plot (**C**) and variable trajectory plot (**D**) of GSE75214 LASSO regression model in dataset. (**E**,**F**) Visualization of the number of genes with the lowest error rate (**E**) and the number of genes with the highest accuracy (**F**) based on SVM algorithm in GSE89408 dataset. (**G**,**H**) LASSO regression analysis in GSE89408 dataset.

Logistic regression models were constructed based on the 87 CGs. The results showed that there were 74 CGs statistically significant in the logistic regression model for the GSE75214 dataset (Supplementary Table S10), and 86 CGs were statistically significant in the logistic regression model for the GSE89408 dataset (Supplementary Table S11). Then, SVM models were constructed based on the selected CGs in the logistic regression models. The number of genes with the lowest error rates (Figure 6A,E) and the highest accuracy rates (Figure 6B,F) were obtained. The results showed that nine genes, which were *DUSP6*, *SPCS3*, *EGR1*, *HIF1A*, *PDIA4*, *NR4A1*, *TTPA*, *LYN*, and *IL1RN*, in the SVM model based on the GSE75214 dataset and 31 genes (Supplementary Table S12) in the SVM model based on GSE89408 had the highest accuracy. The LASSO regression model diagrams (Figure 6C,G) and LASSO variable trajectory diagrams (Figure 6D,H) were drawn for visualization based on the identified CGs in the SVM models. The results showed a total of five CGs (*DUSP6*, *HIF1A*, *PDIA4*, *TTPA* and *LYN*) were identified as IBD-key genes, while 25 CGs (Supplementary Table S13) were identified as RA-key genes.

### 3.6. Identification of Hub Genes and Network Analyses Based on Hub Genes

**Figure 7:**
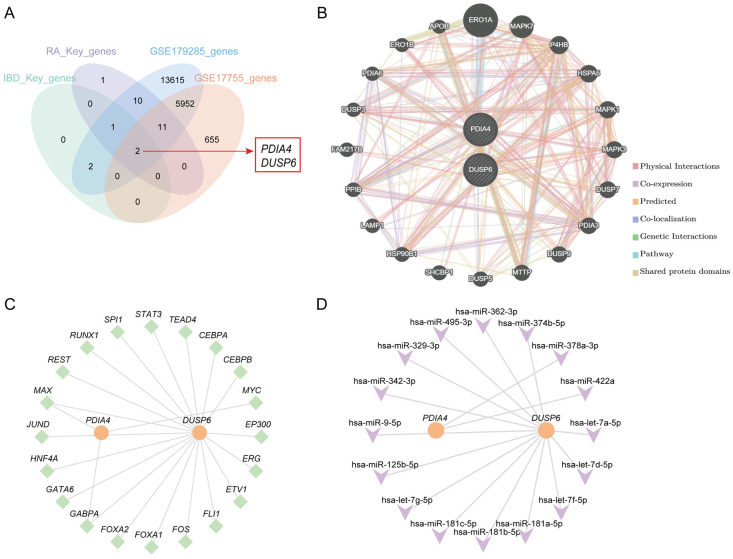
Interaction networks of hub genes. (**A**) Venn diagram presentation of IBD Key genes and RA Key genes, all genes in GSE179285 dataset and all genes in GSE17755 dataset. (**B**) PPI network of hub genes. (**C**) mRNA-TF regulatory network of hub genes. (**D**) mRNA-miRNA regulatory network of hub genes. Orange indicated mRNA, green indicated TF, and purple indicated miRNA.

First, the IBD-key genes, RA-key genes, along with all genes in the GSE179285 and GSE17755 datasets were intersected and visualized using a Venn diagram (Figure 7A). Two genes (*PDIA4* and *DUSP6*) were identified and used as hub genes in subsequent analyses. The PPI network identified 20 proteins with functions similar to PDIA4 and DUSP6 (Figure 7B). Twenty TFs combined with *PDIA4* and *DUSP6* were obtained, and an mRNA-TFs regulatory network was constructed (Figure 7C) (Supplementary Table S14). Finally, 16 miRNAs associated with *PDIA4* and *DUSP6* were identified, and an mRNA-miRNA regulatory network was constructed (Figure 7D) (Supplementary Table S15).

### 3.7. Validation of Diagnostic Models for IBD and RA

**Figure 8:**
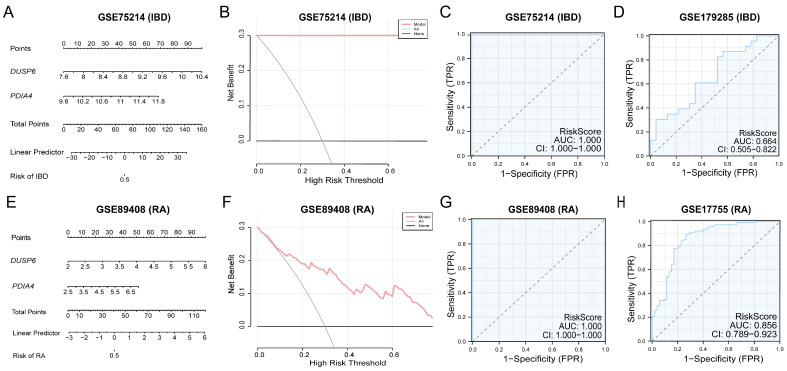
Diagnostic and validation analysis. (**A**) Nomogram of hub genes in GSE75214 in IBD diagnostic model. (**B**) Decision curve of IBD diagnostic model based on hub genes in GSE75214. (**C**) ROC curve of RiskScore in GSE75214. (**D**) ROC curve of RiskScore in GSE179285. (**E**) Nomogram of hub genes in GSE89408 in RA diagnostic model. (**F**) Decision curve of RA diagnostic model based on hub genes in GSE89408. (**G**) ROC curve of RiskScore in GSE89408. (**H**) ROC curve of RiskScore in GSE17755. 0.5 < AUC < 0.7, low accuracy; 0.7 < AUC < 0.9, moderate accuracy; AUC > 0.9, high accuracy.

To verify the value of the diagnostic models for IBD and RA, two nomograms based on *PDIA4* and *DUSP6* were drawn to detail the characteristics and relationships of the hub genes in the GSE75214 and GSE89408 datasets (Figure 8A,E). The results showed that the utility of *DUSP6* expression in the diagnostic models of both IBD and RA was significantly higher than that of the other variables. Whereas, the utility of *PDIA4* expression in the diagnostic models of both IBD and RA was significantly lower than that of other variables.

Based on the hub genes, the role of the diagnostic models for IBD and RA in clinical utility was evaluated using DCA, and the results are displayed through decision curves (Figure 8B,F). The results showed that the model’s curve remained consistently above both all positive and all negative references in a certain range, indicating a greater net benefit and better performance. In addition, the ROC curves based on the risk scores in GSE75214 and GSE89408 showed that the expression level of the risk score had high accuracy among the different groups (AUC > 0.9) (Figure 8C,G). The ROC curves showed certain accuracy among the different groups based on the risk score in GSE179285 (AUC = 0.664) (Figure 8D) and moderate accuracy among the different groups based on the risk score in GSE17755 (AUC = 0.856) (Figure 8H). The risk scores were calculated using the following formula:

GSE179285:riskScore=DUSP6×1.1350+PDIA4×2.7939

GSE17755:riskScore=DUSP6×(−2.5872)+PDIA4×(−2.1597)

### 3.8. ROC Analysis and Validation of Hub Genes

**Figure 9:**
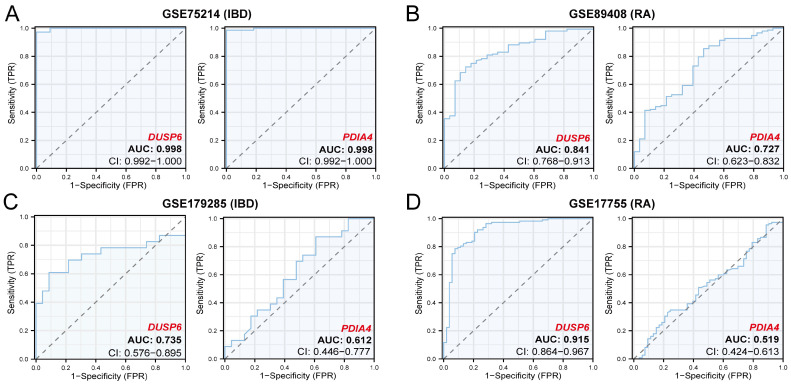
ROC curve analyses of hub genes. (**A**) ROC curves of *DUSP6* and *PDIA4* in GSE75214. (**B**) ROC curves of *DUSP6* and *PDIA4* in GSE89408. (**C**) ROC curves of *DUSP6* and *PDIA4* in GSE179285. (**D**) ROC curves of *DUSP6* and *PDIA4* in GSE17755. 0.5 < AUC < 0.7, low accuracy; 0.7 < AUC < 0.9, moderate accuracy; AUC > 0.9, high accuracy.

ROC curve analysis was performed to evaluate the diagnostic efficacy of the hub genes. The results showed that *DUSP6* and *PDIA4* had high diagnostic accuracy (AUC = 0.998) in distinguishing IBD samples from controls in GSE75214 (Figure 9A). In GSE179285, *DUSP6* had moderate accuracy (AUC = 0.735), whereas *PDIA4* showed limited accuracy (AUC = 0.612) (Figure 9C). For RA classification in GSE89408, both *DUSP6* and *PDIA4* had moderate accuracy (AUC = 0.841 and AUC = 0.727, respectively) (Figure 9B). In GSE17755, *DUSP6* had high accuracy (AUC = 0.915), whereas *PDIA4* had limited accuracy (AUC = 0.519) in distinguishing RA samples from controls (Figure 9D).

### 3.9. Differential Expression Analysis and Validation of DUSP6 and PDIA4

**Figure 10:**
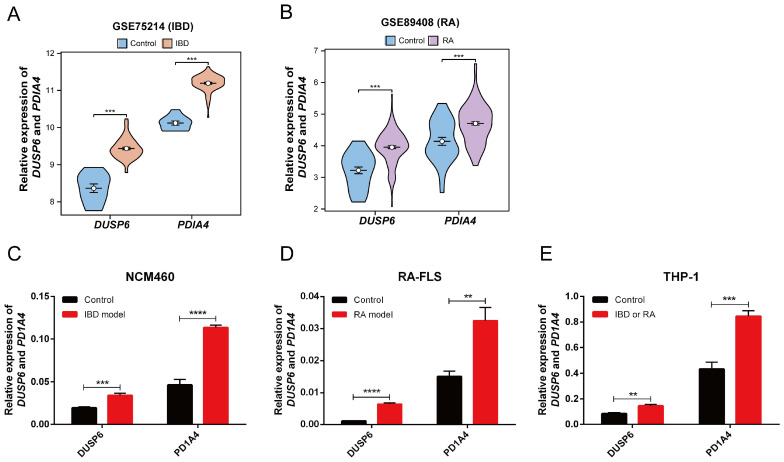
Differential expression analyses of hub genes. (**A**,**B**) Group comparison plots of *DUSP6* and *PDIA4* in GSE75214 and GSE89408. (**C**–**E**) Group comparison plots of *DUSP6* and *PDIA4* in inflammation cell models. ** *p* < 0.01; *** *p* < 0.001; **** *p* < 0.0001.

Differential expression analysis showed that *DUSP6* and *PDIA4* were highly statistically significant in GSE75214 and GSE89408 (Figure 10A,B). However, only *DUSP6* was significant in GSE179285 and GSE17755 (Supplementary Figure S3). Additionally, *DUSP6* and *PDIA4* showed strong statistical significance in the inflammatory cell models derived from NCM460, RA-FLS, and THP-1 (Figure 10C–E).

### 3.10. Immune Infiltration Analysis

**Figure 11:**
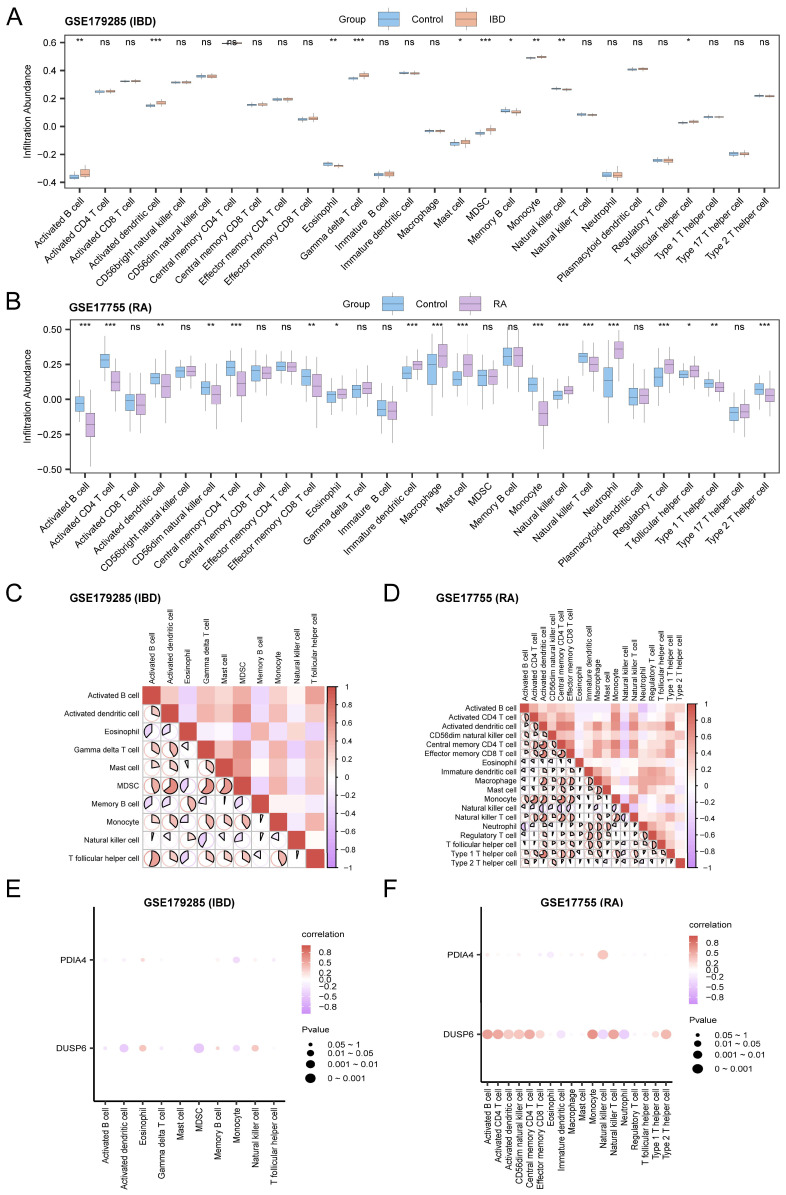
Immune infiltration analysis by ssGSEA algorithm. (**A**,**B**) Relative infiltration abundance of immune cells in IBD and RA. (**C**,**D**) Correlation heatmaps of immune cells infiltration abundance in IBD and RA. (**E**,**F**) Bubble plots of correlation between hub genes and immune cell infiltration abundance in IBD and RA. ns, *p* ≥ 0.05; * *p* < 0.05; ** *p* < 0.01; *** *p* < 0.001. Weak or no correlation, *r* < 0.3; weak correlation, 0.3 < *r* < 0.5; moderate correlation, 0.5 < *r* < 0.8; strong correlation, *r* ≥ 0.8.

The relative infiltration abundances of 28 types of immune cells in IBD and RA are shown in the group comparison diagrams (Figure 11A,B). The results showed that 10 types of immune cells, including activated B cells, activated dendritic cells, and eosinophils, showed statistically significant differences in IBD (Figure 11A), and 18 types of immune cells, including activated B cells, activated CD4 T cells, and activated dendritic cells, showed statistically significant differences in RA (Figure 11B). The correlation results of infiltration abundance of immune cell types showed that most immune cells showed strong correlations, and the strongest positive correlation was observed between activated dendritic cells and MDSCs (*r* = 0.645, *p* < 0.05) in GSE179285 (Figure 11C), and between CD4 T cells and activated dendritic cells (*r* = 0.738, *p* < 0.05) in GSE17755 (Figure 11D). The results of correlations between hub genes and the infiltration abundance of immune cells showed that most immune cells showed strong correlations, among which *DUSP6* and MDSCs had the strongest negative correlation (*r* = −0.485, *p* < 0.05) in GSE179285 (Figure 11E), whereas *DUSP6* and monocytes had the strongest positive correlation (*r* = 0.632, *p* < 0.05) in GSE17755 (Figure 11F).

## 4. Discussion

IBD and RA are chronic inflammatory diseases with similar pathogenesis and often co-occur. Mitochondrial dysfunction plays an important role in the pathogenesis of both diseases, raising the question: Is mitochondrial dysfunction a comorbidity of IBD and RA? This study conducted a series of experiments to address this issue and found a strong association between mitochondrial dysfunction, IBD, and RA with mitochondrial dysfunction. The results identified potential diagnostic biomarkers and molecular mechanisms associated with mitochondrial dysfunction in IBD and RA, providing assistance in developing targeted treatment strategies and improving patient prognosis.

Our differential genes expression analysis revealed that a total of 182 IBD-MDRDEGs and 187 RA-MDRDEGs, which were obtained after DEGs intersected with MDRGs, were intricately linked to mitochondrial dysfunction and differentially expressed in IBD and RA. By further combining the results of the WGCNA, 87 CGs were identified, underscoring the relevance between mitochondrial dysfunction and the comorbidity of IBD and RA. The findings from WGCNA supported the significance of these genes, as they were clustered within modules that correlated with disease traits. This emphasizes the potential of these genes as biomarkers for early detection of IBD and RA.

GO enrichment analysis revealed that these CGs were involved in critical biological processes, such as cytokine production and immune responses, reinforcing their potential as therapeutic targets. KEGG enrichment analysis revealed that these CGs were significantly enriched in biological pathways, such as the AGE-RAGE signaling pathway and NF-κB signaling pathway, which play crucial roles in inflammatory diseases through mutual regulation [36,37]. These findings suggested that mitochondrial dysfunction might lead to the production of cellular inflammatory factors, immune response disorders, and activation of inflammation-related biological processes, such as AGE-RAGE and NF-κB signaling pathway, and thus play a role in the pathogenesis of IBD and RA. Our study emphasizes the importance of normal mitochondrial function in maintaining immune homeostasis and suppressing inflammation and suggests that restoring mitochondrial function may be a potential strategy for managing IBD and RA. Moreover, GSVA was performed to elucidate the complex biological pathways involved in IBD and RA, including the immune response and PECAM1 interaction pathways, providing further insights into their pathogenesis [38].

Importantly, our study identified two hub genes associated with mitochondrial dysfunction in IBD and RA: *DUSP6* and *PDIA4*. DUSP6, a member of the larger family of protein tyrosine phosphatases, is a key regulator of the ERK/MAPK signaling pathway that modulates mucosal immune and inflammatory responses, and has emerged as a critical player in the pathogenesis of both IBD and RA [39,40,41]. In IBD, *DUSP6* serves as a critical node in the regulation of intestinal inflammation and epithelial homeostasis [42]. Studies have shown that *DUSP6* deletion in the colonic epithelium protects against inflammation by suppressing ERK/MAPK signaling, thereby reducing the production of pro-inflammatory cytokines and mitigating mucosal damage [43]. Similarly, in RA, *DUSP6* deletion has been shown to protect against autoimmune arthritis by reducing inflammation and joint damage through the suppression of ERK/MAPK signaling, which attenuates the activation of synovial fibroblasts and immune cells [40]. Furthermore, studies have demonstrated that the *DUSP6* inhibitor (E/Z)-BCI hydrochloride can modulate oxidative stress and exert anti-inflammatory effects in RA by activating the Nrf2 antioxidant signaling axis and suppressing the NF-κB pathway [40]. PDIA4, a member of the protein disulfide isomerase (PDI) family, plays a critical role in protein folding, the endoplasmic reticulum stress response, and cellular homeostasis [44]. Studies have implicated *PDIA4* in the pathogenesis of both IBD and RA, particularly through its role in oxidative stress regulation, inflammatory signaling, and immune cell activation [45,46,47].

Given the significant roles of *DUSP6* and *PDIA4* in both IBD and RA, could they serve as predictive biomarkers or therapeutic targets for these diseases, and how do they affect their pathogenesis? To explore these questions, diagnostic models for IBD and RA, PPI networks, and regulatory networks were established using *DUSP6* and *PDIA4*. The ROC curves for the *DUSP6* and *PDIA4* diagnostic models showed high accuracy (AUC ≈ 1) in the training sets for both IBD and RA, and showed a moderate accuracy in their validation sets (AUC were 0.664 and 0.856, respectively). These results suggest that *PDIA4* and *DUSP6* may serve as potential diagnostic biomarkers for mitochondrial dysfunction in IBD and RA. To further validate these findings, the diagnostic efficacy of *DUSP6* and *PDIA4* was validated in both IBD and RA, yielding consistent results.

The PPI network identified 20 proteins with functions similar to those of PDIA4 and DUSP6. We were particularly interested in MAPK1, MAPK3, MAPK7, and LAMP1. MAPK1, MAPK3, and MAPK7 are members of MAPK (mitogen-activated protein kinase) family that play a central role in regulating inflammation and immune responses. In IBD, dysregulated MAPK signaling contributes to the disruption of intestinal epithelial barrier function, release of pro-inflammatory cytokines, exacerbation of intestinal inflammation, and promotion of intestinal fibrosis by activating fibroblast [48]. MAPK signaling pathways are mainly involved in the occurrence and development of RA by regulating synovial inflammation and promoting the proliferation and invasion of synovial fibroblasts [49]. LAMP1 (lysosomal-associated membrane protein 1) is widely present in the lysosomal and endosomal membranes. It plays an important role in maintaining lysosomal integrity, regulating autophagy, participating in immune and inflammatory responses, and improving mitochondrial dysfunction. These functions allow LAMP1 to play an important role in various inflammatory diseases, including IBD and RA [50,51].

Twenty TFs associated with *PDIA4* and *DUSP6* were identified, among which the transcription factors MYC-associated factor X (MAX) and GA-binding protein alpha (GABPA) may simultaneously regulate *PDIA4* and *DUSP6*. MAX, a core component of the MYC/MAX/MAD network, forms heterodimers with MYC or MAD proteins to regulate genes involved in cell proliferation, differentiation, and metabolic reprogramming, particularly in cancer and inflammatory diseases [52,53]. GABPA, a member of the ETS family, plays a critical role in mitochondrial biogenesis by regulating TFAM expression. It also modulates immune cell activation, metabolic adaptation, and cell cycle progression [54]. Both MAX and GABPA contribute to disease pathogenesis by influencing metabolic rewiring, immune regulation, and tissue remodeling. Research has shown that MAX and GABPA may bind to consensus CCCTC-binding factor sites, suggesting that they may regulate the expression of downstream genes through synergistic effects [55]. Combined with our results, MAX and GABPA may synergistically regulate the expression of *DUSP6* and *PDIA4*.

Additionally, immune infiltration analysis indicated significant differences in the infiltration of various immune cell types in IBD and RA, suggesting their crucial role in the pathogenesis of these diseases. The analysis revealed that certain immune cell types, such as activated B cells and dendritic cells, exhibited marked variations in abundance, consistent with previous findings and highlighting the importance of immune cell dynamics in chronic inflammatory conditions [56]. The differential infiltration patterns of immune cells in IBD and RA and correlations between hub genes and infiltration abundance of immune cells suggest that therapeutic strategies targeting these immune mechanisms could be beneficial for both IBD and RA. These results provide insight for future studies aimed at developing targeted immunotherapies and improving the outcomes of IBD and RA.

Our study has some limitations that must be acknowledged. First, the relatively small sample size may have affected the generalizability of the findings. Therefore, larger cohorts are needed to validate the results. Second, we only conducted basic differential expression validation of hub genes using cell models, lacking in-depth analysis of key pathways, molecular mechanisms, and in vivo experiments. Third, our research mainly relied on dataset analyses and cell models of IBD or RA rather than patient samples from individuals with both diseases. Future studies should incorporate in vivo and in vitro models to validate key pathways and molecular mechanisms. Ideally, clinical samples from patients with IBD and RA and animal models of combined IBD and RA should be used to further investigate the link between these two diseases, as well as between the two diseases and mitochondrial dysfunction.

## 5. Conclusions

In summary, through an integrative network analysis, we have delineated a shared molecular architecture between IBD and RA rooted in mitochondrial dysfunction. We identified *PDIA4* and *DUSP6* as central hub genes within this architecture, which not only demonstrate high diagnostic accuracy in prediction models but also correlate significantly with disease-specific immune cell infiltration profiles.

These findings posit *PDIA4* and *DUSP6* as robust candidate biomarkers and imply that their common regulatory networks may harbor shared therapeutic targets for managing the IBD-RA comorbidity.

Looking forward, this study opens several methodological avenues. The network framework established here serves as a foundation for future multi-omics integration (e.g., proteomics, metabolomics) to capture a more comprehensive pathological landscape. Technically, the roles of *PDIA4* and *DUSP6* warrant validation in cell-type-specific contexts using single-cell RNA sequencing and in experimental models of comorbidity. Furthermore, applying deep learning models to this network could predict novel drug targets or reposition existing therapies. Ultimately, translating these findings will require validation in clinical cohorts of patients with both IBD and RA, moving beyond the analysis of separate disease datasets toward a truly integrated comorbidity model.

## Figures and Tables

**Table 1:** The datasets information on GEO database.

Dataset	Platform	Species	Source	Disease	Control	Group
GSE75214	GPL6244	*Homo sapiens*	colon	74 (IBD)	11	Experiment
GSE179285	GPL6480	*Homo sapiens*	colon	23 (IBD)	23	Validation
GSE89408	GPL11154	*Homo sapiens*	synovial	152 (RA)	28	Experiment
GSE17755	GPL1291	*Homo sapiens*	Peripheral blood	112 (RA)	53	Validation

## Data Availability

The original contributions presented in this study are included in the article/Supplementary Materials. Further inquiries can be directed to the corresponding author.

## Supplementary Materials

The following supporting information can be downloaded at: https://www.mdpi.com/article/10.3390/cimb48010089/s1.
