# Supplementary material for: Exploring the Shared Diagnostic Biomarkers and Molecular Mechanisms Related to Mitochondrial Dysfunction in Inflammatory Bowel Disease and Rheumatoid Arthritis
Source: Curr Issues Mol Biol. 2026 Jan 16;48(1):89. doi: 10.3390/cimb48010089 (PMC12840288; doi:10.3390/cimb48010089)
Supplement: Supplementary file 1 [file cimb-48-00089-s001.zip › cimb-4082505-supplementary/Supplementary Figures/Supplementary Figure Legends.pdf]

Supplementary Figure S1: Data set cleaning. (A-B) Boxplot of GSE75214 distribution before (A) and after (B) standardized processing. (C-D) Boxplot of GSE179285 distribution before (C) and after (D) standardized treatment. (E-F) Boxplot of GSE89408 distribution before (E) and after (F) standardized treatment. (G-H) Boxplot of GSE17755 distribution before (G) and after (H) standardized treatment. (Related description: See Main Text, Page 3, Section “2.1 Data Acquisition and Preprocessing”.)

Supplementary Figure S2: WGCNA for GSE75214 and GSE89408. (A) Venn diagrams of 2703 MDRGs and the genes contained in the black, blue, pink, purple, and red modules of GSE75214 (IBD). (B) Venn diagrams of 2703 MDRGs and the genes contained in the blue, brown, magenta, pink, red, and turquoise modules of GSE89408 (RA). (Related description: See Main Text, Page 7-8, Section “3.2 WGCNA and the Acquisition of CGs of IBD and RA”.)

Supplementary Figure S3: Differential expression analyses of hub genes in validation sets. (A) Group comparison plots of *DUSP6* and *PDIA4* in GSE179285 (IBD). (B) Group comparison plots of *DUSP6* and *PDIA4* in GSE17755 (RA). (Related description: See Main Text, Page 16, Section “3.9 Differential Expression Analysis and Validation of *DUSP6* and *PDIA4*”.)
