# Supplementary material for: Exploring the Shared Diagnostic Biomarkers and Molecular Mechanisms Related to Mitochondrial Dysfunction in Inflammatory Bowel Disease and Rheumatoid Arthritis
Source: Curr Issues Mol Biol. 2026 Jan 16;48(1):89. doi: 10.3390/cimb48010089 (PMC12840288; doi:10.3390/cimb48010089)
Supplement: Supplementary file 1 [file cimb-48-00089-s001.zip › cimb-4082505-supplementary/Supplementary Tables/Supplementary Table S14-The list of mRNA-TF interaction network nodes..pdf]

Supplementary Table S14: mRNA-TF interaction network nodes.

mRNA	TF
DUSP6	EP300
DUSP6	ERG
DUSP6	ETV1
DUSP6	FLI1
DUSP6	FOS
DUSP6	FOXA1
DUSP6	FOXA2
DUSP6	GABPA
DUSP6	GATA6
DUSP6	HNF4A
DUSP6	JUND
DUSP6	MAX
DUSP6	REST
DUSP6	RUNX1
DUSP6	SPI1
DUSP6	STAT3
DUSP6	TEAD4
DUSP6	CEBPA
DUSP6	CEBPB
PDIA4	GABPA
PDIA4	MAX
PDIA4	MYC

TF：Transcription factor。
