# Supplementary material for: Exploring the Shared Diagnostic Biomarkers and Molecular Mechanisms Related to Mitochondrial Dysfunction in Inflammatory Bowel Disease and Rheumatoid Arthritis
Source: Curr Issues Mol Biol. 2026 Jan 16;48(1):89. doi: 10.3390/cimb48010089 (PMC12840288; doi:10.3390/cimb48010089)
Supplement: Supplementary file 1 [file cimb-48-00089-s001.zip › cimb-4082505-supplementary/Supplementary Tables/Supplementary Table S15-The list of mRNA-miRNA interaction network nodes..pdf]

Supplementary Table S15: mRNA-miRNA interaction network nodes.

mRNA	miRNA
DUSP6	hsa-let-7a-5p
DUSP6	hsa-let-7d-5p
DUSP6	hsa-let-7f-5p
DUSP6	hsa-miR-181a-5p
DUSP6	hsa-miR-181b-5p
DUSP6	hsa-miR-181c-5p
DUSP6	hsa-let-7g-5p
DUSP6	hsa-miR-125b-5p
DUSP6	hsa-miR-9-5p
DUSP6	hsa-miR-342-3p
DUSP6	hsa-miR-329-3p
DUSP6	hsa-miR-495-3p
DUSP6	hsa-miR-362-3p
DUSP6	hsa-miR-374b-5p
PDIA4	hsa-miR-378a-3p
PDIA4	hsa-miR-422a
