# Supplementary material for: Exploring the Shared Diagnostic Biomarkers and Molecular Mechanisms Related to Mitochondrial Dysfunction in Inflammatory Bowel Disease and Rheumatoid Arthritis
Source: Curr Issues Mol Biol. 2026 Jan 16;48(1):89. doi: 10.3390/cimb48010089 (PMC12840288; doi:10.3390/cimb48010089)
Supplement: Supplementary file 1 [file cimb-48-00089-s001.zip › cimb-4082505-supplementary/Supplementary Tables/Supplementary Table S7-Results of GO and KEGG enrichment analysis..pdf]

Supplementary Table S7: Results of GO and KEGG enrichment analysis.

ONTOLOGY	ID	Description	GeneRatio	BgRatio	p-Value	p-Adjust	q-Value
BP	GO:0001819	positive regulation of cytokine production	20/87	475/18800	3.76322E-14	1.01155E-10	6.3539E-11
BP	GO:1901652	response to peptide	18/87	491/18800	8.55629E-12	1.14997E-08	7.22331E-09
BP	GO:0043434	response to peptide hormone	16/87	404/18800	4.53234E-11	4.06097E-08	2.55083E-08
CC	GO:0009897	external side of plasma membrane	12/87	455/19594	7.80467E-07	0.00011698	8.79549E-05
CC	GO:0005788	endoplasmic reticulum lumen	10/87	311/19594	1.19367E-06	0.00011698	8.79549E-05
CC	GO:0062023	collagen-containing extracellular matrix	10/87	429/19594	2.02803E-05	0.001324982	0.000996227
MF	GO:0005178	integrin binding	7/87	156/18410	9.07875E-06	0.003395452	0.002752295
MF	GO:0044389	ubiquitin-like protein ligase binding	8/87	317/18410	0.000127019	0.023752568	0.019253418
MF	GO:0005126	cytokine receptor binding	7/87	272/18410	0.000303638	0.037853486	0.030683377
KEGG	hsa05140	Leishmaniasis	8/67	77/8164	1.80215E-07	3.83859E-05	2.80757E-05
KEGG	hsa04933	AGE-RAGE signaling pathway in diabetic complications	7/67	100/8164	1.5962E-05	0.001099229	0.000803983
KEGG	hsa04064	NF-kappa B signaling pathway	7/67	104/8164	2.06428E-05	0.001099229	0.000803983

GO，Gene Ontology；BP，Biological Process；CC，Cellular Component；MF，Molecular Function；KEGG，Kyoto Encyclopedia of Genes and Genomes。
