# Supplementary material for: Exploring the Shared Diagnostic Biomarkers and Molecular Mechanisms Related to Mitochondrial Dysfunction in Inflammatory Bowel Disease and Rheumatoid Arthritis
Source: Curr Issues Mol Biol. 2026 Jan 16;48(1):89. doi: 10.3390/cimb48010089 (PMC12840288; doi:10.3390/cimb48010089)
Supplement: Supplementary file 1 [file cimb-48-00089-s001.zip › cimb-4082505-supplementary/Supplementary Tables/Supplementary Table S8-Results of GSVA (IBD_Control) for GSE75214..pdf]

Supplementary Table S8: Results of GSVA(IBD/Control) for GSE75214.

ID	logFC	AveExpr	t	P-Value	adj. P-Val	B
BIOCARTA BLYMPHOCYTE PATHWAY	1.086172621	0.046111097	5.685576261	1.35E-07	2.07E-06	7.247100428
BIOCARTA LYM PATHWAY	1.069226471	0.041347868	6.042953428	2.75E-08	7.92E-07	8.767228352
REACTOME METALLOTHIONEINS BIND METALS	-1.041295844	-0.026405892	-5.851139682	6.49E-08	1.26E-06	7.94597446
REACTOME INTERLEUKIN 21 SIGNALING	1.038005015	0.040891208	5.899808991	5.22E-08	1.15E-06	8.1532091
BIOCARTA MONOCYTE PATHWAY	1.030473157	0.021501228	5.397133595	4.73E-07	4.80E-06	6.053496512
REACTOME INTERLEUKIN 35 SIGNALLING	1.020795903	0.048473912	6.235205473	1.15E-08	5.11E-07	9.60195752

REACTOME INTERLEUKIN 6 SIGNALING	1.017441307	0.043626153	6.176088145	1.50E-08	5.83E-07	9.344096321
REACTOME FCGR ACTIVATION	1.007543273	0.058561014	6.554865716	2.63E-09	2.41E-07	11.01320097
BIOCARTA TCRA PATHWAY	1.007243762	0.061357055	5.271441201	8.08E-07	6.98E-06	5.54357534
BIOCARTA TCYTOTOXIC PATHWAY	1.005599257	0.049734767	5.244067623	9.08E-07	7.50E-06	5.433387488
BIOCARTA GRANULOCYTES PATHWAY	0.991887814	0.028610878	5.423615931	4.22E-07	4.35E-06	6.161746882
BIOCARTA THELPER PATHWAY	0.980366556	0.032992373	4.931396093	3.34E-06	1.83E-05	4.197860768
WP MAMMARY GLAND DEVELOPMENT PATHWAY INVOLUTION STAGE 4 OF 4	0.974107425	0.05806938	7.465525588	3.46E-11	2.04E-08	15.16260327
WP IMMUNE RESPONSE TO TUBERCULOSIS	0.970935947	0.04451258	6.643830389	1.73E-09	2.08E-07	11.41070999

WP OMEGA 9 FATTY ACID SYNTHESIS	0.970103197	0.079863347	7.622573184	1.62E-11	1.50E-08	15.89333347
PID INTEGRIN4 PATHWAY	0.968817008	0.045991753	6.119894715	1.94E-08	6.63E-07	9.099952134
WP PLATELET MEDIATED INTERACTIONS WITH VASCULAR AND CIRCULATING CELLS	0.968739952	0.038482817	5.622784647	1.78E-07	2.47E-06	6.984597394
REACTOME PECAM1 INTERACTIONS	0.960559819	0.044168472	6.561416284	2.55E-09	2.41E-07	11.04240297
KEGG MEDICUS REFERENCE BETA OXIDATION	-0.952858071	-0.022658884	-6.134238049	1.82E-08	6.36E-07	9.162179269
WP GLYCOLYSIS IN SENESCENCE	0.951063225	0.051826802	6.524867021	3.02E-09	2.61E-07	10.87960744

GSVA，Gene Set Variation Analysis。
