# Supplementary material for: Exploring the Shared Diagnostic Biomarkers and Molecular Mechanisms Related to Mitochondrial Dysfunction in Inflammatory Bowel Disease and Rheumatoid Arthritis
Source: Curr Issues Mol Biol. 2026 Jan 16;48(1):89. doi: 10.3390/cimb48010089 (PMC12840288; doi:10.3390/cimb48010089)
Supplement: Supplementary file 1 [file cimb-48-00089-s001.zip › cimb-4082505-supplementary/Supplementary Tables/Supplementary Table S9-Results of GSVA (RA_Control) for GSE89408..pdf]

Supplementary Table S9: Results of GSVA(RA/Control) for GSE89408.

ID	logFC	AveExpr	t	P-Value	adj. P-Val	B
KEGG MEDICUS REFERENCE HEDGEHOG SIGNALING PATHWAY	-0.925074598	-0.069653636	-8.61444925	2.48E-15	1.23E-12	24.30080954
KEGG MEDICUS REFERENCE N GLYCAN PRECURSOR BIOSYNTHESIS ALG6 TO OST	0.878488816	0.040286283	7.937584333	1.64E-13	3.06E-11	20.23716047
BIOCARTA PROTEASOME PATHWAY	0.87223827	0.032332166	8.010902004	1.05E-13	2.24E-11	20.6696878
KEGG MEDICUS PATHOGEN HTLV 1 TAX TO NFY MEDIATED TRANSCRIPTION	0.845895552	0.048793658	7.034570146	3.38E-11	9.96E-10	15.08430325
PID P38 GAMMA DELTA PATHWAY	-0.837293037	-0.076595312	-9.786794771	1.27E-18	3.78E-15	31.65686946
REACTOME FOLDING OF ACTIN BY CCT TRIC	0.836283192	0.040027752	7.351812975	5.41E-12	2.55E-10	16.8558426
KEGG MEDICUS PATHOGEN HIV VPR TO CDC25 CELL CYCLE G2M	0.829316855	0.047822917	7.613533312	1.15E-12	9.29E-11	18.34972626

BIOCARTA EIF2 PATHWAY	0.825077071	0.059162037	8.750853104	1.05E-15	6.24E-13	25.13764779
REACTOME SYNTHESIS OF PIPS AT THE LATE ENDOSOME MEMBRANE	0.824445341	0.082493728	9.441762918	1.22E-17	1.82E-14	29.45616052
REACTOME ADENYLATE CYCLASE ACTIVATING PATHWAY	-0.794498087	-0.044203396	-7.536895562	1.82E-12	1.26E-10	17.90936807
KEGG MEDICUS REFERENCE ANTIGEN PROCESSING AND PRESENTATION BY MHC CLASS II MOLECULES	0.791720714	0.050922526	6.043392735	7.64E-09	7.53E-08	9.861004458
BIOCARTA MONOCYTE PATHWAY	0.779920329	0.041556759	7.032159514	3.43E-11	1.00E-09	15.07101403
WP HEDGEHOG SIGNALING PATHWAY WP47	-0.769113554	-0.061059711	-7.717592035	6.20E-13	5.95E-11	18.95139618
REACTOME IRAK4 DEFICIENCY TLR2 4	0.768864935	0.051968682	8.381096638	1.07E-14	3.55E-12	22.88255255
REACTOME REGULATION OF IFNG SIGNALING	0.759455798	0.00821358	7.383999816	4.48E-12	2.26E-10	17.03802651

KEGG PROTEIN EXPORT	0.752738305	0.02366903	7.850746017	2.78E-13	3.76E-11	19.72743772
BIOCARTA EGFR SMRTE PATHWAY	-0.736981377	-0.039534696	-7.570214713	1.49E-12	1.08E-10	18.10052747
KEGG MEDICUS REFERENCE FSHR GNAS PKA SIGNALING PATHWAY	-0.736736782	-0.048508737	-8.125452395	5.21E-14	1.41E-11	21.34934215
KEGG MEDICUS REFERENCE PTH PTH1R PKA SIGNALING PATHWAY	-0.732142891	-0.047093303	-7.877869473	2.36E-13	3.53E-11	19.8863443
BIOCARTA RAB PATHWAY	0.72293196	0.053353379	7.868893215	2.49E-13	3.53E-11	19.83372503

GSVA，Gene Set Variation Analysis.
