# Supplementary figures and images for: Exploring the Shared Diagnostic Biomarkers and Molecular Mechanisms Related to Mitochondrial Dysfunction in Inflammatory Bowel Disease and Rheumatoid Arthritis
Source: Curr Issues Mol Biol. 2026 Jan 16;48(1):89. doi: 10.3390/cimb48010089 (PMC12840288; doi:10.3390/cimb48010089)

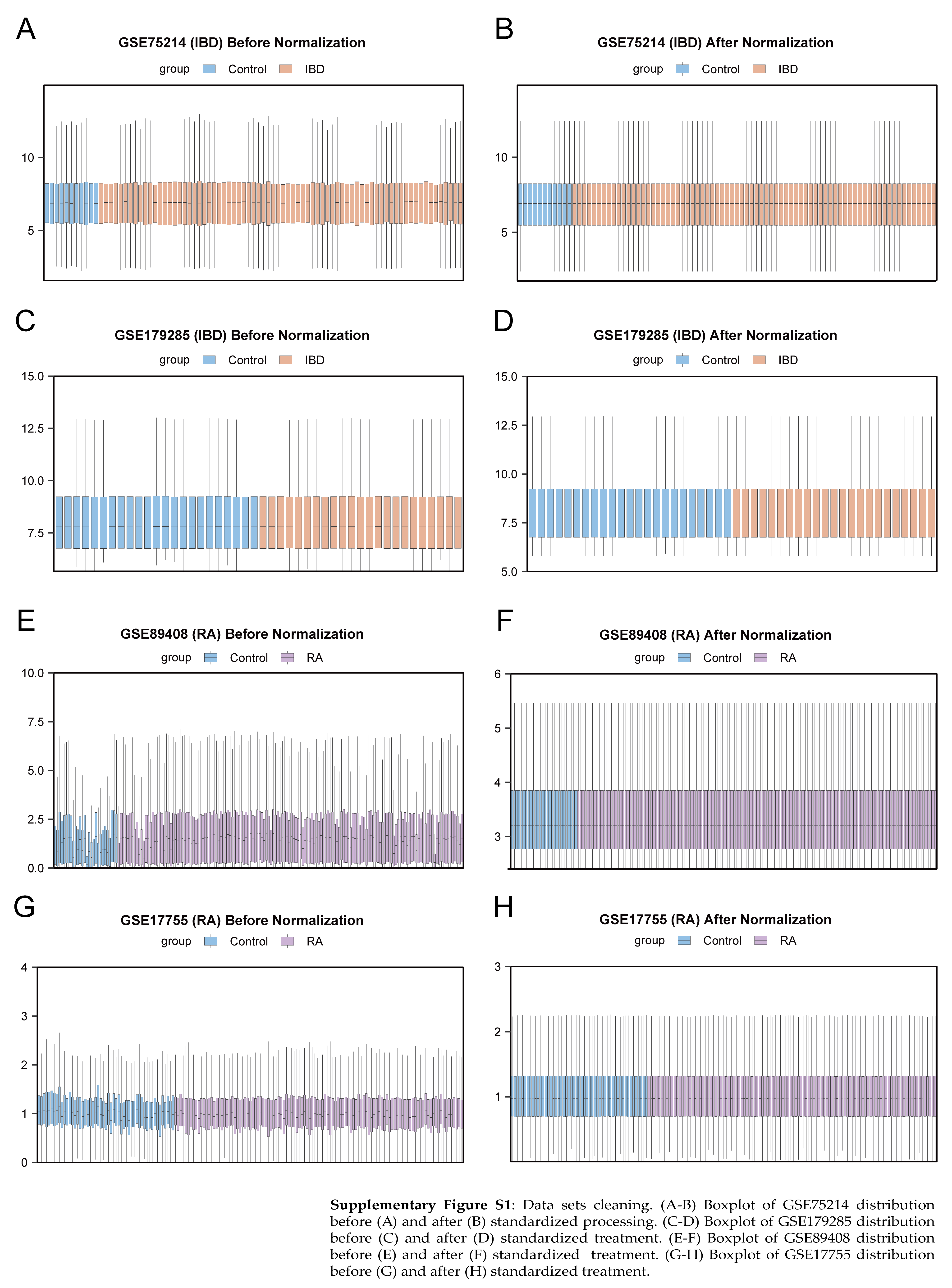

Supplement: Supplementary file 1 [file cimb-48-00089-s001.zip › cimb-4082505-supplementary/Supplementary Figures/Supplementary Figure S1. Data sets cleaning.tif]

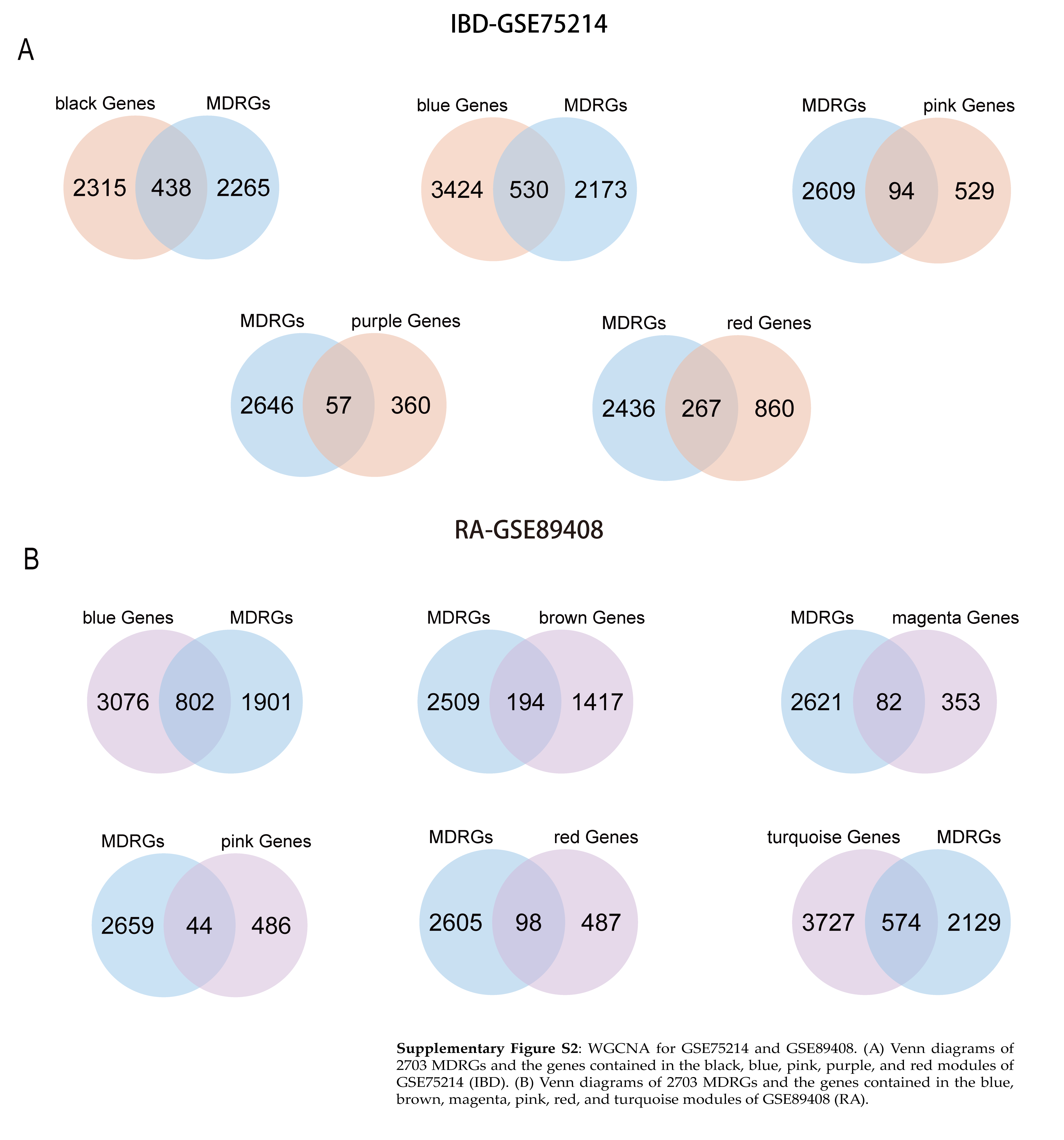

Supplement: Supplementary file 1 [file cimb-48-00089-s001.zip › cimb-4082505-supplementary/Supplementary Figures/Supplementary Figure S2. WGCNA for GSE75214 and GSE89408.tif]

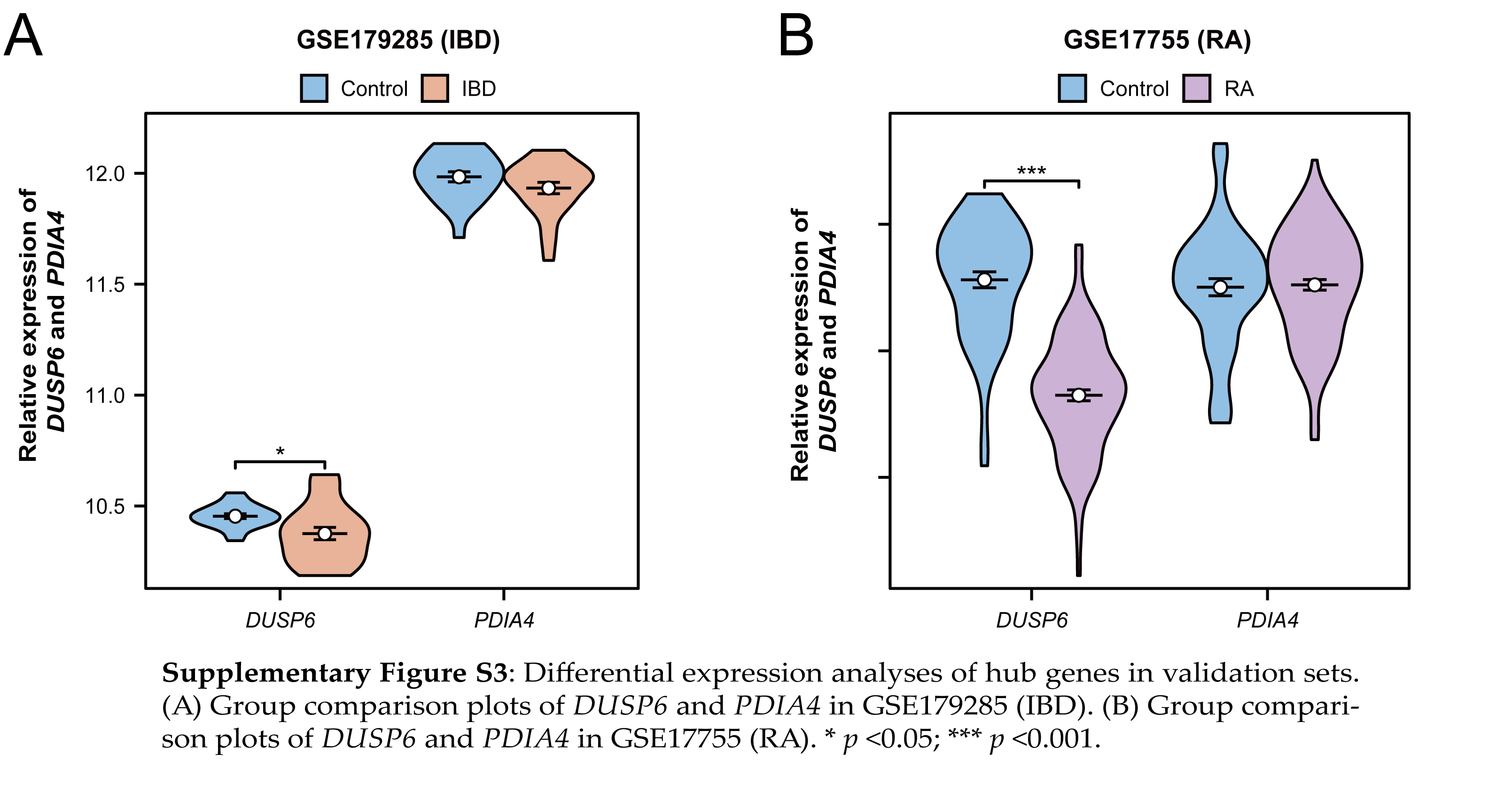

Supplement: Supplementary file 1 [file cimb-48-00089-s001.zip › cimb-4082505-supplementary/Supplementary Figures/Supplementary Figure S3. Differential expression analyses of hub genes in validation sets..tif]
